# Influence of Turn-Taking in a Two-Person Conversation on the Gaze of a Viewer

**DOI:** 10.1371/journal.pone.0071569

**Published:** 2013-08-12

**Authors:** Lotta Hirvenkari, Johanna Ruusuvuori, Veli-Matti Saarinen, Maari Kivioja, Anssi Peräkylä, Riitta Hari

**Affiliations:** 1 Brain Research Unit, O.V. Lounasmaa Laboratory, Aalto University School of Science, Espoo, Finland; 2 School of Social Sciences and Humanities, University of Tampere, Tampere, Finland; 3 Department of Social Research, University of Helsinki, Helsinki, Finland; University of Muenster, Germany

## Abstract

In natural conversation, the minimal gaps and overlaps of the turns at talk indicate an accurate regulation of the timings of the turn-taking system. Here we studied how the turn-taking affects the gaze of a non-involved viewer of a two-person conversation. The subjects were presented with a video of a conversation while their eye gaze was tracked with an infrared camera. As a control, the video was presented without sound and the sound with still image of the speakers. Turns at talk directed the gaze behaviour of the viewers; the gaze followed, rather than predicted, the speakership change around the turn transition. Both visual and auditory cues presented alone also induced gaze shifts towards the speaking person, although significantly less and later than when the cues of both modalities were available. These results show that the organization of turn-taking has a strong influence on the gaze patterns of even non-involved viewers of the conversation, and that visual and auditory cues are in part redundant in guiding the viewers’ gaze.

## Introduction

Behind the apparent ease of conversation, whether a formal or a more casual one, lies a tight organization of speaking turns [1]. The coordinated turn-taking system enables the fluency and continuity of natural conversation and entails regulation of the timings of turns at talk. Over different languages and cultures, the conversation participants share an ability to exchange the speakership in tens of milliseconds and, for most of the time, without overlaps [2]. Most gaps between the turns are shorter than 400 ms, about one third of them even less than 200 ms [3], meaning that they are too short to be achieved only by reacting to the end of the previous speaker’s utterance. Instead, the conversation participants have to predict when the previous speaker is going to finish her turn of talk [4]. The generic structure of turn-taking that defines the opportunities for turn transition has been suggested to guide the conversation participants in this prediction [1]. Moreover, motor-cortex oscillations entrained to the syllable rhythm have been proposed to enable the fine-tuning of the timing of speakership change [4], [5].

According to the organization of turn-taking, one party talks at a time, and the change of the turn is allowed in transition-relevance places [1]. The participants of the conversation cue the phase of the current utterance and the proximity of the next transition-relevance place in different ways, both nonverbally and with signs embedded in their speech. The syntactic, pragmatic and prosodic cues in speech signal when the current utterance reaches its completion; the more cues are clustered together, the stronger is the signal for turn transition [6].

Gaze, as an important nonverbal gesture, has a role in coordinating conversation and turn-taking as well as in informing about the person’s target of attention. The speaker’s gaze direction may signal that the turn is approaching completion: the speaker makes eye contact with the listener around turn exchange, whereas the eye contact is less common when the speaker is still continuing the turn [7]–[9]. Furthermore, a speaker of a “first pair part”, i.e., an action (such as a question) that makes relevant a response from the co-participant (such as an answer), can deploy gaze shift to the participant after the completion of the first pair part, as means for pursuing the relevant response [10]. The speaker is also more likely to gaze at the listener during phrase-boundary pauses of a long turn, probably for checking whether the listener is still following [8]. Accordingly, the listener often produces accompaniment signals that indicate comprehension during speaker’s phrase-boundary-pause gazes [8], [11]. The observations about listeners’ gaze behaviour around the turn exchange are less consistent, as reports exist about keeping the eye contact for a while [7], [9] as well as about looking away immediately or even in advance of starting one’s turn [8]. Gaze withdrawal by both the speaker and the listener at the end of utterance can signal an understanding that the sequence including the utterance is being closed [10].

In addition to face-to-face conversation, gaze coordination occurs in many other situations involving social interaction. For example when subjects are listening to a previously recorded description of the same TV scene they are viewing, their eye movements follow the eye movements of the speaker with an average delay of 2 s [12]. Similarly, eye movements of two persons discussing a shared scene are coupled as an indication of their joint attention to the same events and locations on the screen [13]. The tight coupling of gaze patterns is further increased when the subjects share background knowledge about the topic of conversation [13]. The coordinated eye and body movements have been suggested to embody the similarity of the cognitive processes under similar cognitive constraints during social interaction [14].

In the present study we showed a group of volunteers a short video of a real conversation to find out how people follow a two-party conversation with their gaze. As a control, the same video was presented without sound and the same soundtrack with a still frame captured from the original video. To be able to assess the effects of turn transitions and the turn-transition cues to followers of a natural conversation, we developed quantitative methods for analysing gaze behaviour of a group of subjects. This experimental setup with passive viewing of a conversation presented from video enabled us to expose each subject to an identical natural conversation, to evaluate the effect of the verbal and nonverbal cues separately, and to focus our analysis on any conversational events of interest to assess the similarity of the viewers’ gaze behaviour. Although our subjects were non-involved viewers of the conversation, their gaze was expected to be informative of the most attention-capturing aspects of the conversation.

We hypothesized that accumulation of the syntactic, pragmatic and prosodic cues of turn transition would prompt a shift of gaze even in a viewer of the conversation. We further expected to see synchronous gaze behaviour between subjects, directed by the organization of turn-taking. We also studied the timing of the gaze patterns with respect to turn transitions.

## Materials and Methods

### Subjects

Nineteen Finnish-speaking young adults (10 males, 9 females; aged 19–33 years, mean 27.4) with normal or corrected-to-normal vision volunteered in the main experiment. Nine subjects participated in the two control experiments, video with no sound (6 males, 3 females; aged 22–36 years, mean 28.3) or still image with sound (5 males, 4 females; aged 25–37 years, mean 31.2).

### Ethics Statement

The subjects gave their written informed consent prior to the experiment after the course of the study had been explained to them. The study had prior approval by the Ethics Committee of the Hospital District of Helsinki and Uusimaa.

### Stimulus

In the main experiment, we presented an audiovisual stimulus: a 5.5-min video clip of two women (conversation participant 1 and 2, later referred as ‘P1’ and ‘P2’) having a conversation. As a control, (i) the same video was shown without sound (later referred as ‘video only’) and (ii) the same soundtrack was played with only a still frame captured from the original video (later referred as ‘sound only’). The criteria for choosing the original video clip were that *(i)* the viewer would be guaranteed an access to the facial and posture orientation of the participants of the conversation and *(ii)* to the topic talked about, *(iii)* the speaker should change rather rapidly and one participant should not hold the turn for long, and *(iv)* the scene should be clean of any other possibly interesting items.

**Figure 1:**
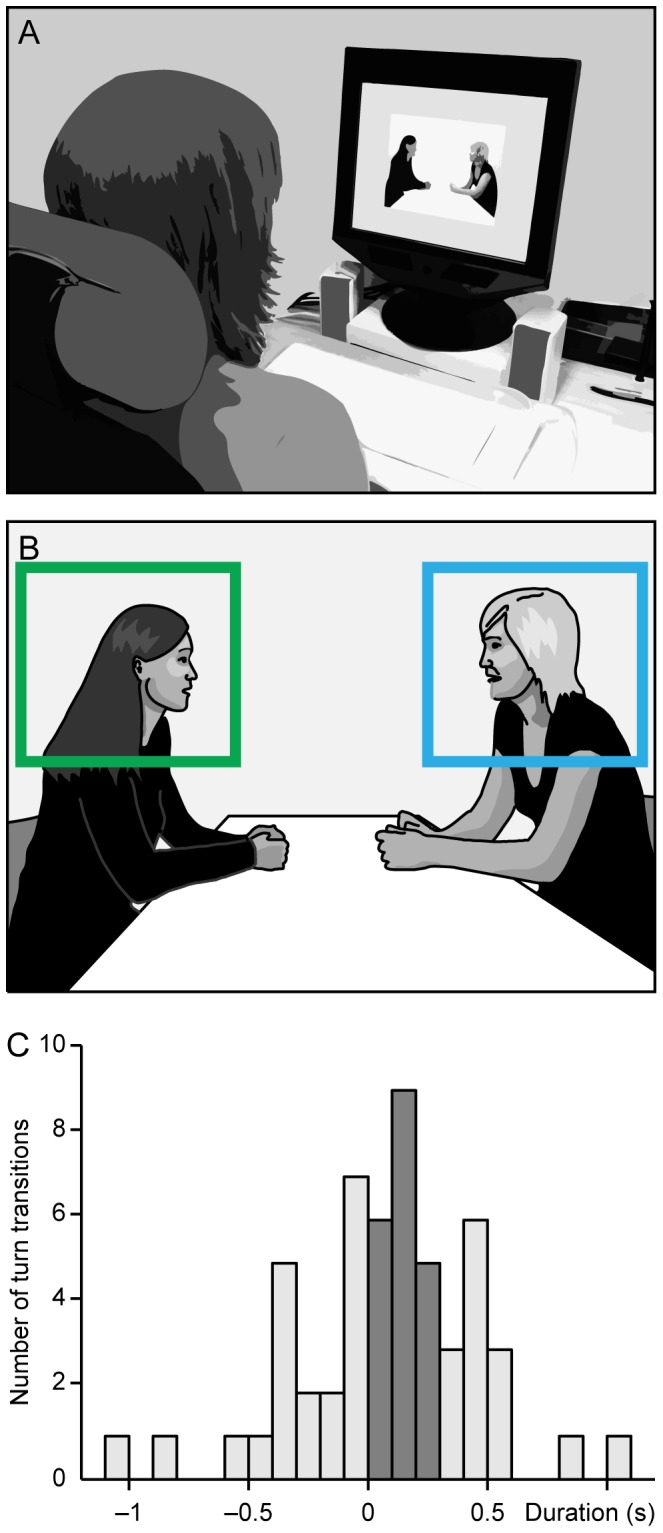
Experimental setup and stimulus. **A.** The subject is viewing conversation from a screen with a built-in eye tracker. **B.** A line-art rendering of the original stimulus video. The regions of interest, covering the heads of both conversation participants, are shown as green and blue squares (ROI 1 for P1 and ROI 2 for P2, respectively). **C.** Turn-transition durations in the conversation. The fast turn transitions (fTT) are shown in dark grey.

The video was presented with ClearView 2.7.1 software (Tobii Technology AB, Sweden) on a grey background, and its size was 20 deg×16 deg (16 deg×12 deg for 4/19 subjects because of different display resolution settings) when viewed from the distance of 60 cm. The sound was presented through stereo loudspeakers, located on both sides of the screen (see Fig. 1A for the experimental setup and Fig. 1B for stimulus video). The subjects were asked to attentively follow the conversation, without any other instructions.

### Conversation Analysis

The conversation on the stimulus video was analysed for three cue combinations that signal turn completion. In the order from the weakest to the strongest cue for turn-taking [6], the cue combinations were syntactic only (S), syntactic+pragmatic (SPra), and syntactic+pragmatic+prosodic (SPraPro). All timings of the cues were referred to the end of the word.

An example of the transcription and cue types is given below. The participants have been talking about arranging surprise parties, and P1 has told about her success in making such arrangements. The extract starts when she has just closed her anecdote saying ”so I think I was the main master of ceremony in this”, after which she pauses for a moment and finishes with the utterance at line 1. Following the utterance, a speaker change occurs at line 3.

[1∶14∶0]

1 P1: … ne onnistu kyllä hyvin ne juhlat.

They succeeded in fact really well those parties.

**S S SPra S SPraPro**

**start end**

2 (.)

3 P2: mt. Mh >Kuinka paljon< siel oli ihmisiä.

Mt. Mh >How many<people were there.

[1∶17∶3]

The example illustrates syntactic (S), pragmatic (Pra), and prosodic (Pro) cues for turn transition. Syntactic cues mark places where the utterance could be syntactically complete notwithstanding the preceding context. Pragmatic cues mark places where the utterance can be understood as complete in its conversational context. Prosodic cues refer to places where the intonation of the utterance sounds as if the speaker is finishing his/her turn of talk [6]. In Finnish, a strong cue for closing the utterance is falling intonation, which is marked with a full stop in the transcript. All three types of cues typically cluster at the transition-relevance place, predicting turn transition [6]. In the conversation used as our stimulus, the mean (± standard deviation) distances from each cue combination type to the turn transition were 6.3±5.5 s, 4.5±6.4 s and 1.3±4.0 s for the S, SPra, and SPraPro cue combinations, respectively.

In addition to the turn-transition cues, routinely annotated in conversation analysis, we also defined the ends and the starts of speech, followed or preceded by a minimum of ∼0.5 s of silence. We defined ‘fast turn transition’ (fTT) as an event type in which the speaker change (from the end of one speaker’s speech to the start of the other’s) occurred in less than 300 ms and without overlap of the two speakers; altogether 41% (22/54; see Fig. 1C) of all turn transitions occurring during the conversation belonged to the fTT category.

The 5.5-min conversation contained altogether 34 S, 134 SPra and 94 SPraPro events, 126 Starts, 126 Ends, and 22 fTTs.

P1 spoke alone for 33% of the video duration, P2 spoke alone for 44% of the time, and both participants spoke simultaneously for 5%. Both remained silent for 18% of the video duration.

### Eye Tracking

Subjects’ gaze position on the screen was recorded during the video presentation by Tobii 1750 eye tracker (Tobii Technology AB, Sweden) and ClearView 2.7.1 software, with a sampling frequency of 50 Hz. The eye tracker was calibrated before the experiment by asking the subjects to fixate at five points that covered the screen. The tracking was done binocularly and based on video-oculography by dark-pupil–corneal-reflection method; the infrared camera and the infrared light sources were integrated into the computer screen.

### Data Analysis

As the faces of the conversation participants in the stimulus video stayed relatively still during the whole recording, positions of two regions of interest (ROIs), one ROI for each face, were defined as stationary rectangles (see Fig. 1B).

The gaze data were further analysed in Matlab 7 (MathWorks). Gaze direction changes were defined as gaze moving from one ROI to the other in less than 70 ms, later referred as ‘ROI change’. This limit was chosen because a 10-deg saccade lasts for about 40 ms [15] but may, because of the sampling frequency of 50 Hz, be detected only after 60 ms.

For the analysis of the group-level effects, we computed the mean ROI change rate over all subjects separately for all different conversational events, in 200-ms windows across a 5-s (from –2 to 3 s) epoch centered on each event; the same analysis was made for the main experiment and for the two control conditions. For comparison, the baseline level of ROI change rate was estimated in the same manner around “events” placed equidistantly once every 6 s throughout the video.

The effect of visual and auditory cues to the subjects’ responses to turn transitions was assessed by comparing the cumulative distributions of ROI changes around fTTs in the three experimental conditions with Kolmogorov-Smirnov test in Matlab.

## Results

**Figure 2:**
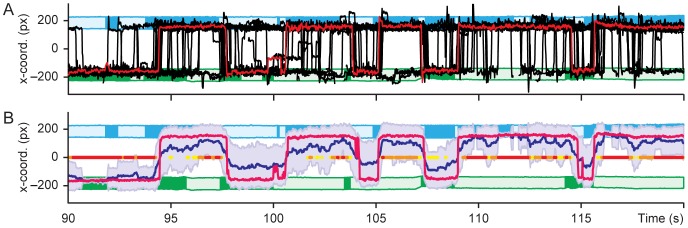
Horizontal gaze locations (x-coordinates) during a 30-s epoch of the conversation. The ROIs indicate the locations of the speakers’ heads (see Fig. 1B), dark blue and green denoting speech and light colours silence. **A.** The red trace shows the gaze of one representative subject and the black lines the gazes of each individual subject. **B.** Median (red line), mean (dark blue line), and standard error of mean (grey belt) of all subjects’ gaze x-coordinates. The time points significantly differing from random distribution of subjects between ROIs are shown along the x  = 0 line with yellow, orange, and red dots, corresponding to statistical significance levels of 0.05, 0.01, and 0.001, respectively (binomial test).

Figure 2A shows a 30-s sample of the eye tracking data, with the x-coordinate of the gaze location, i.e. the gaze position at horizontal plane, plotted as a function of time for all subjects. The ROI locations are shown on the background with blue and green horizontal blocks, with the dark colours denoting speech and the light colours silence. Figure 2B shows the same epoch with the mean (blue) and the median (red) of the x-coordinates.

The data of Fig. 2 show a clear trend for the gaze to turn to the subject who is speaking. In the main experiment, the subjects’ gaze was in the ROIs for on average 92% of the video duration; 40% of the gazes were directed to ROI 1 and 52% to the ROI 2, whereas only 3% of the gazes landed outside the ROIs and 5% of the data were missing. The speaking person attracted statistically significantly more gazes than the person remaining silent (*p*<10^–9^; see Table 1). Subjects changed their gaze from one ROI to another 103±9 (mean ± SEM) times during the 5.5-min long measurement, that is on average once every 3 s (the turn transitions occurred on average once every 6 s).

**Table 1:** Proportions of gazes (in %) to different targets in the screen during speech of P1 (33% of the total time), P2 (44%), both (5%), silence (18%) and during the whole stimulus presentation (total).

Sound+video	P1	P2	Both	Silence	Total
**ROI 1**	69	15	54	45	40
**ROI 2**	22	78	37	47	52
**other areas**	4	3	3	4	3
**missing**	5	5	5	4	5
**Video only**	**P1**	**P2**	**Both**	**Silence**	**Total**
**ROI 1**	68	21	61	48	43
**ROI 2**	23	72	33	45	49
**other areas**	7	5	4	5	6
**missing**	2	2	2	2	2
**Sound only**	**P1**	**P2**	**Both**	**Silence**	**Total**
**ROI 1**	61	18	50	42	38
**ROI 2**	24	67	37	44	47
**other areas**	9	8	8	8	9
**missing**	6	7	5	6	6

The statistical significance of the similarity vs. randomness of the subjects’ gaze behaviour is indicated with coloured dots along the x  = 0 line of Fig. 2B. Since the viewer could, at any moment, gaze either participant P1 or P2, in random viewing the viewers’ gazes would be binomially distributed, with *p*  = 0.5, between the two ROIs. The colour codes refer to *p*-values (0.05 yellow, at least 14 out of 19 subjects; 0.01 orange, at least 16 subjects; and 0.001 red, at least 17 subjects) for obtaining the observed sample from such an underlying distribution. We noted that for 68% of the total time at least 14 out of the 19 subjects viewed the same ROI.

**Figure 3:**
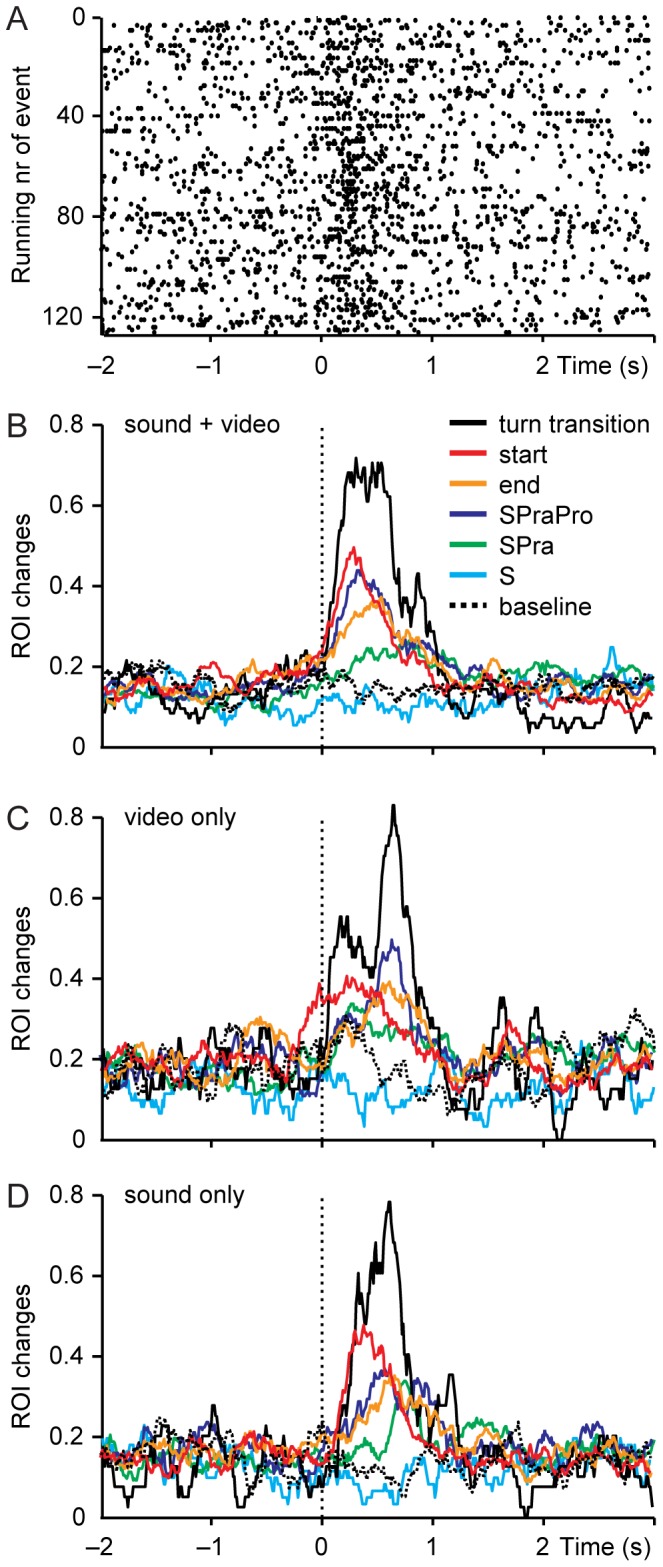
ROI changes around conversational events. **A.** Single ROI changes relative to each start-event. Each vertical line of dots represents a 5-s epoch around one start of speech in conversation, and a single dot represents one ROI change of one subject. **B.** Mean ROI change rate in main experiment around all conversational events: ROI changes per second as a function of time relative to each event, normalized according to number of subjects. **C.** Mean ROI change rate in video-only condition. **D.** Mean ROI change rate in sound-only condition.

The effects of various turn completion cues and of other events in the conversation were assessed by examining the gaze-direction changes around each cue. Fig. 3A shows the gaze-direction changes of all subjects towards the ROI of the conversation participant who starts to speak at time 0. ROI changes occur throughout the interval from 2 s before to 3 s after the start of speech, but they clearly cluster within the first second after the speech onset. The panels 3B–3D show the ROI change rates as a function of time in the main experiment, video-only and sound-only conditions. In the main experiment, the rate of ROI changes started to rise at the time of the event and peaked 0.3–0.7 s after the event, depending on the event type (Fig. 3B). For the turn-transition cues, the stronger the cue combination, the more ROI changes it evoked. Similar behaviour was seen in video-only and sound-only conditions (Fig. 3C and 3D). Since the subjects could not hear the speech-embedded syntactic, pragmatic and prosodic cues in the video-only condition, the ROI changes that seem to be related to the turn-transition cues actually have to reflect the strong interdependence between the turn transitions and turn-transition cues.

**Figure 4:**
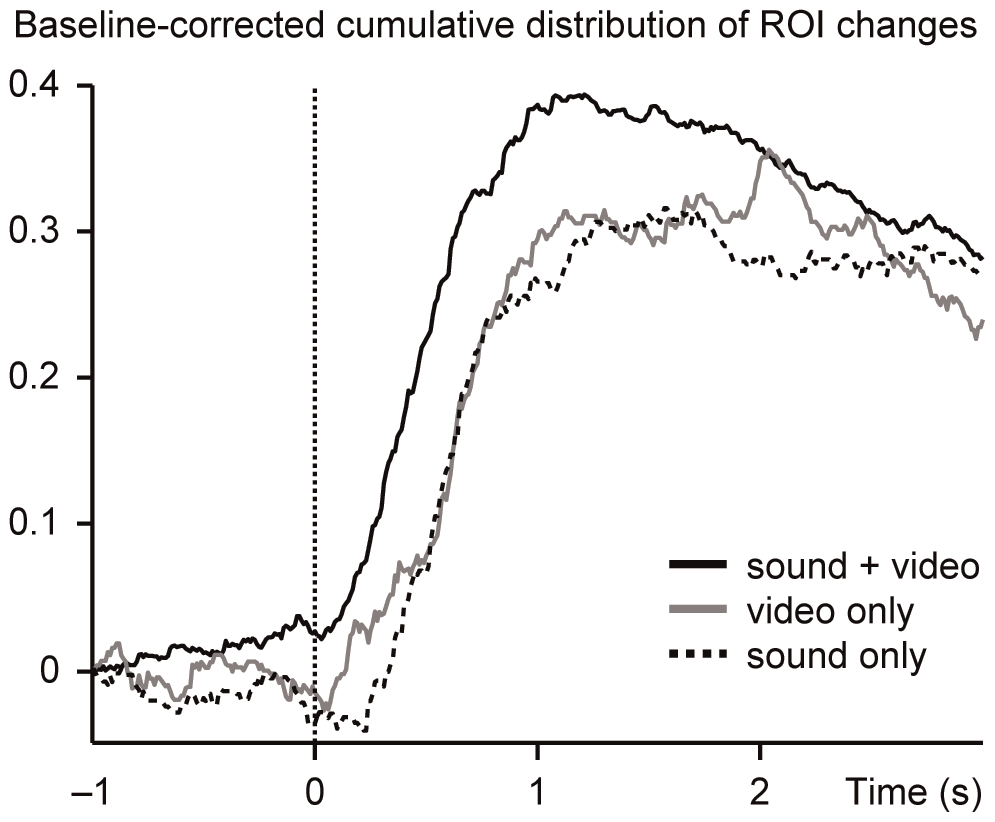
Baseline-corrected cumulative distributions of ROI changes around fast turn transitions. Cumulative distributions of ROI changes, with baseline cumulative distributions of ROI changes subtracted, during a time interval from –1 s to 3 s relative to the fast turn transitions. The black, the grey and the dashed line represent the main experiment, video-only and sound-only conditions.

Figure 4 shows the baseline-corrected cumulative distributions of ROI changes around fTTs in the main experiment and in the two control conditions; the baseline level of ROI changes was subtracted from each distribution which explains their decrease after the maximum values about 1.2 s after the fTT. The distributions for both control conditions lag the distribution for the main experiment by about 200 ms, and the total number of ROI changes is about 20% smaller in the control conditions than in the main experiment. The distributions (without baseline correction) for both the video-only and sound-only conditions differed statistically significantly from the distribution for the main experiment (p  = 0.017 and p  = 0.0047; Kolmogorov-Smirnov test), whereas the control conditions did not differ from each other (p  = 0.29).

## Discussion

Our goal was to understand, by tracking the eye gaze, how uninvolved viewers follow a conversation between two other persons. The gaze patterns across our 19 subjects showed similarity that differed statistically significantly from random viewing. Turns at talk directed the subjects’ gaze so that the current speaker received most of the gazes. The time spent looking at the current speaker was on average 74%, which closely resembles the behaviour of participants in a two-party conversation in which the listener looks at the speaker on average 75% of the time [16]. The organization of turn-taking is known to direct the gaze of conversation participants [7]–[9], and our results extend the effect to the gaze of passive viewers.

The fTTs induced gaze-target changes also in the control experiments when either only the verbal (sound only) or only the nonverbal (video only) cues of turn transition were present. In human speech, speech-sound envelope and mouth opening are highly correlated, and they are both modulated at 2–7 Hz [17] corresponding to the syllable rhythm. This redundancy of auditory and visual speech, especially at the frequencies suggested to underlie the timing of turn-taking [4], could explain why both auditory and visual cues alone guided the gaze of the viewers in a manner resembling the effect these cues had together. However, the viewers reacted slower to the turn transitions when cues of only one modality were available, no matter whether they were auditory or visual. Based on our results it therefore seems likely that neither verbal nor nonverbal cues can result in the same conversation-following accuracy as is achieved when both cues are simultaneously present. Contrary to our hypothesis, the accumulation of verbal cues of turn transition did not play a crucial role in guiding the gaze of a non-involved viewer.

Although we found–as we had expected–that the stronger the cue for turn transition, the more gaze-direction changes it elicits, very similar behaviour was seen also in the video-only condition, in which the subjects could not hear the verbal cues. Thus this result had to arise from the strong temporal correlation of turn transitions and the different turn-transition cues. In addition, because the cue strength is inversely related to the temporal distance to turn transition, it is obvious that the stronger is the cue for turn transition, the more its effects are contaminated by the effects of the close-by turn transition. Consequently, we had to abandon any further attempts to analyse the effects of different verbal cues of turn transition on the viewer’s gaze behaviour. Our results suggest a need for further research on visual cueing–by means of participants’ gaze as well as by body posture and kinesics–of turn transition, and its coordination with the verbal cueing.

We did not observe anticipation of turn transition in the viewers’ gaze behaviour; instead, most of the gaze-target changes occurred about 0.5 s after fast turn transition, and about 0.3 s after start of speech. This lag equals the lag of a saccade towards a simple auditory target [18], implying that the viewer’s attention shifted to a conversation participant as soon as she started speaking. In this sense the viewers’ gaze behaviour was similar to the gaze patterns in two-party conversations, where the prediction of turn transitions is not accompanied by anticipatory gaze behaviour; the gaze rather serves a different purpose in turn-taking [19]. In a two-party conversation, the listener usually keeps the eye contact to the speaker at least until the speakership has changed [7], [9]. If the viewers of a two-party conversation would follow the same pattern as they do when they are actually involved in the conversation, then they would be expected to look at the speaker until the turn has ended, which is in line with our results. However, Foulsham et al. [20] recently reported the viewers to anticipate turn transitions by 150 ms. In comparison with our stimulus presenting free, relaxed conversation with only a couple of questions, their video contained a more tense situation with debating and persuasion. In such a situation the viewers may be more eager to follow the reactions of the other conversation participant and thus anticipate the turn-takings, which would explain the different gaze timings in these two studies.

Our experimental setup is in line with the currently dominant tradition of social neuroscience, where the effects of social stimuli to an uninvolved viewer are assessed. However, this “spectator stance” has been criticized (see e.g. [21], [22]), as it neglects the real inter-subject interaction that is a crucial part of real-life social situations, and enables the participants themselves to affect how the situation unfolds. Our subjects could not participate in the conversation but were just following it passively, which could have impaired their tuning into the natural conversation rhythm. Passive listening differs from participating in a conversation e.g. when it comes to breathing: in conversation, the breathing rhythm of the speaker and the listener synchronizes around a turn exchange [23], but not if the listener is not able to respond [4]. Our subjects’ gaze behaviour may thus have been less tightly linked to the turn-taking than if the task would have been more engaging, or if the subjects would have actually taken part in the conversation. However, our findings are well in line with earlier results about two-person conversation, implying that the experimental setup was natural enough to trigger gaze patterns that resemble behaviour in real-life conversations. Furthermore, although our subjects were just spectators of the conversation, their gaze behaviour was expected to be informative of the most attention-capturing aspects of the conversation. Our results of the highly similar and replicable reactions to turn transitions indicate that the organization of turn-taking has a strong influence on the gaze patterns of even non-involved viewers of the conversation.

## References

[1] Sacks H, Schegloff EA, Jefferson G (1974) Simplest systematics for organization of turn-taking for conversation. Language 50: 696–735.

[2] Stivers T, Enfield NJ, Brown P, Englert C, Hayashi M, et al (2009) Universals and cultural variation in turn-taking in conversation. Proc Natl Acad Sci USA 106: 10587–10592. 19553212 10.1073/pnas.0903616106 PMC2705608

[3] Wilson TP, Zimmerman DH (1986) The structure of silence between turns in two-party conversation. Discourse Proc 9: 375–390.

[4] Wilson M, Wilson TP (2005) An oscillator model of the timing of turn-taking. Psychon Bull & Rev 12: 957–968. 16615316 10.3758/bf03206432

[5] Scott SK, McGettigan C, Eisner F (2009) A little more conversation, a little less action-candidate roles for the motor cortex in speech perception. Nat Rev Neurosci 10: 295–302. 19277052 10.1038/nrn2603 PMC4238059

[6] Ford CE, Thompson SA (1996) Interactional units in conversation: Syntactic, intonational, and pragmatic resources for the management of turns. In: Ochs E, Schegloff EA, Thompson SA, editors. Interaction and grammar. New York: Cambridge University Press. 134–184.

[7] Novick DG, Hansen B, Ward K (1996) Coordinating turn-taking with gaze. Proc ICLP-96: 1888–1891.

[8] Kendon A (1967) Some functions of gaze-direction in social interaction. Acta Psychol 26: 22–63. 10.1016/0001-6918(67)90005-4 6043092

[9] Levine MH, Sutton-Smith B (1973) Effects of age, sex, and task on visual behavior during dyadic interaction. Devel Psychol 9: 400–405.

[10] Rossano F (2012) Gaze behavior in face-to-face interaction. PhD Thesis, Radboud University Nijmegen, Nijmegen.

[11] Bavelas JB, Coates L, Johnson T (2002) Listener responses as a collaborative process: The role of gaze. J Communic 52: 566–580.

[12] Richardson DC, Dale R (2005) Looking to understand: The coupling between speakers' and listeners' eye movements and its relationship to discourse comprehension. Cogn Sci 29: 1045–1060. 21702802 10.1207/s15516709cog0000_29

[13] Richardson DC, Dale R, Kirkham NZ (2007) The art of conversation is coordination - Common ground and the coupling of eye movements during dialogue. Psychol Sci 18: 407–413. 17576280 10.1111/j.1467-9280.2007.01914.x

[14] Shockley K, Richardson DC, Dale R (2009) Conversation and Coordinative Structures. Topics Cogn Sci 1: 305–319. 10.1111/j.1756-8765.2009.01021.x 25164935

[15] Carpenter RHS (1988) Saccades. In: Carpenter RHS, editor. Movement of the Eyes. London: Pion. 69–111.

[16] Argyle M, Ingham R (1972) Gaze, mutual gaze and proximity. Semiotica 6: 32–49.

[17] Chandrasekaran C, Trubanova A, Stillittano S, Caplier A, Ghazanfar AA (2009) The natural statistics of audiovisual speech. PLoS Comput Biol 5: e1000436. 19609344 10.1371/journal.pcbi.1000436 PMC2700967

[18] Zambarbieri D, Schmid R, Magenes G, Prablanc C (1982) Saccadic responses evoked by presentation of visual and auditory targets. Exp Brain Res 47: 417–427. 7128709 10.1007/BF00239359

[19] Argyle M, Cook M (1974) Gaze and Mutual Gaze. Cambridge: Cambridge University Press.

[20] Foulsham T, Cheng JT, Tracy JL, Henrich J, Kingstone A (2010) Gaze allocation in a dynamic situation: Effects of social status and speaking. Cognition 117: 319–331. 20965502 10.1016/j.cognition.2010.09.003

[21] Di Paolo EA, De Jaegher H (2012) The interactive brain hypothesis. Front Hum Neurosci 6: 163. 22701412 10.3389/fnhum.2012.00163 PMC3369190

[22] Risko EF, Laidlaw K, Freeth M, Foulsham T, Kingstone A (2012) Social attention with real versus reel stimuli: toward an empirical approach to concerns about ecological validity. Front Hum Neurosci 6: 143. 22654747 10.3389/fnhum.2012.00143 PMC3360477

[23] McFarland DH (2001) Respiratory markers of conversational interaction. J Speech Lang Hear R 44: 128–143. 10.1044/1092-4388(2001/012) 11218097

